# Transcriptome Analysis Reveals the Anti-cancerous Mechanism of Licochalcone A on Human Hepatoma Cell HepG2

**DOI:** 10.3389/fnut.2021.807574

**Published:** 2021-12-20

**Authors:** Jun Wang, Bo Wei, Kiran Thakur, Chu-Yan Wang, Ke-Xin Li, Zhao-Jun Wei

**Affiliations:** ^1^School of Biological Food and Environment, Hefei University, Hefei, China; ^2^School of Food and Biological Engineering, Hefei University of Technology, Hefei, China; ^3^School of Biological Science and Engineering, North Minzu University, Yinchuan, China

**Keywords:** Licochalcone A, HepG2 cells, transcriptome analysis, transcription factor, the MAPK signaling pathway, the FoxO signaling pathway

## Abstract

Hepatocellular carcinoma is a malignancy with a low survival rate globally, and there is imperative to unearth novel natural phytochemicals as effective therapeutic strategies. Licochalcone A is a chalcone from *Glycyrrhiza* that displayed various pharmacological efficacy. A globally transcriptome analysis was carried out to reveal the gene expression profiling to explore Licochalcone A's function as an anti-cancer phytochemical on HepG2 cells and investigate its potential mechanisms. Altogether, 6,061 dysregulated genes were detected (3,414 up-regulated and 2,647 down-regulated). SP1 was expected as the transcription factor that regulates the functions of most screened genes. GO and KEGG analysis was conducted, and the MAPK signaling pathway and the FoxO signaling pathway were two critical signal pathways. Protein-protein interaction (PPI) network analysis based on STRING platform to discover the hub genes (MAPK1, ATF4, BDNF, CASP3, etc.) in the MAPK signaling pathway and (AKT3, GADD45A, IL6, CDK2, CDKN1A, etc.) the FoxO signaling pathway. The protein level of essential genes that participated in significant pathways was consistent with the transcriptome data. This study will provide an inclusive understanding of the potential anti-cancer mechanism of Licochalcone A on hepatocellular, signifying Licochalcone A as a promising candidate for cancer therapy.

## Introduction

Liver cancer is the sixth malignant neoplasm and is the fourth leading cause of cancer death worldwide (1). Hepatocellular carcinoma (HCC) affects 75% of patients with liver cancer (2). The pathogenesis of HCC is considered a complicated progression mainly influenced with complex factors (3). The environmental factors, eating habits, smoking, and alcohol consumption are related to the pathogenesis of HCC (4). In recent decades, the main principles of HCC treatment are operation, radiotherapy, and chemotherapy. However, all these strategies have been inadequate due to toxic side effects, drug resistance, and limited effectiveness (5, 6). Hence, there is an urgent need to explore potential HCC therapeutic methods.

The genus *Glycyrrhiza*, also known as licorice, includes two important species: *Glycyrrhiza uralensis Fisch*. and *Glycyrrhiza glabra L*. which are widely spread in Asia, Europe, America, and Africa (7). The *Glycyrrhiza* genus is widely used for food and medicinal purposes. The sweetness of *Glycyrrhiza* makes it widely used as a confectionary and condiments. It also contains various nutrition, such as saponins and chalcones, which enabled it to display antioxidant, antiproliferative, anti-inflammation, and immunoregulatory activities (8). It contains diverse phytochemicals such as saponins and chalcones. Licochalcone A (LCA), a chalcone extracted from licorice roots, demonstrated potential pharmacological properties in asthma, obesity, and radical scavenging and anti-cancer activity (9). Notably, multiple studies reported that LCA could block the metastasis in multiple cancer cells as prostate cancer cells PC-3, gastric cancer cells MKN-28 and MKN-45, osteosarcoma cells HOS and MG-63, lung cancer cells A549 and H460, breast cancer cell MDA-MB-231, and hepatoma cell HepG2 (9–14) *in vitro*. Nevertheless, the transcriptomics-based mechanism has not been fully elucidated.

RNA sequencing (RNA-seq) is a high-throughput sequencing developed in the next-generation sequencing technology context (15). RNA-seq can directly sequence the cDNA translated by the RNA, and the attained readings can then be matched to a reference genome to accomplish the transcriptional map. RNA-seq is an effective method for in-depth transcriptome exploration and is extensively applied in the biomedical field (16). Compared with other transcriptome methods such as DNA microarrays, RNA-seq has the advantage of high reproducibility, high accuracy, and low signal interference (17).

According to our previous study, LCA caused HepG2 cytotoxicity with IC_50_ (65.96 μM) after 24 h, induced cell cycle arrest and apoptosis, and elevated the cellular ROS levels in HepG2 cells (9). In the current study, the transcriptome profiling in LCA-treated Human Hepatoma cells HepG2 was conducted by RNA-seq technology to understand the underlying metabolic and signaling pathways. A progressed recognition of mechanism of action exerted by LCA at the omics levels will promote innovatively and effective therapeutic strategies to strive against HCC.

## Materials and Methods

### Materials

LCA (purity 91.12%) was extracted from *Glycyrrhiza*, which was obtained from Xingjiang province, China (9). The culture medium defined fetal bovine serum, and antibiotics (penicillin and streptomycin) were acquired from the Hyclone lab (Logan, UT, USA). The cell strain of human hepatoma cell HepG2 was obtained from Shanghai Wei atlas biological technology co., LTD. Antibodies were procured from Cell Signaling Technology (MA, USA).

### Cell Culture Conditions and Treatments

HepG2 was cultured in the culture medium supplemented with 10% fetal bovine serum and 1% antibiotics at 37°C in a humidified atmosphere of 5% CO_2_ (18). The cells (3 × 10^5^) were seeded on 100 × 20 mm dishes and incubated overnight. After the treatment with 70 μM of LCA for 24 h, the cells were required. Non-exposed cells were used as controls.

### Total RNA Isolation and Detection

The total RNA was extracted by the RNA simple Total RNA Kit (TIANGEN, Beijing, China). Each treatment had three duplicates (Control 1, Control 2, and Control 3; Treat A1, Treat A2, and Treat A3). The total RNA purity and integrity were evaluated by Agilent Technologies 2,100 Bioanalyzer. Then the integrated and purified RNA was quantified and examined spectrophotometrically. The samples were considered acceptable when the total amount of RNA was > 15 μg, the concentration was > 200 ng/μL, and the A_260_/A_280_ ratio ranged between 1.8 and 2.2.

### cDNA Library Construction and Sequencing

The RNA-Seq process was completed at the Shanghai Personal Biotechnology (Shanghai, China) (19). For RNA-seq, the cDNA libraries were constructed using the mRNA-Seq sample preparation kit (Illumina, San Diego, USA). Briefly, the mRNA with polyA structure in the total RNA was enriched by poly-T oligo attached magnetic beads (Invitrogen, CA, USA). And then, the mRNA was regimented into small segments at a higher temperature. Then, these short pieces were used as templates to synthesize the first-strand cDNA with random hexamers primers and reverse transcriptase (Invitrogen, CA, USA). Subsequently, second-strand cDNA synthesis was completed with RNase H and DNA polymerase I. After the second strand was synthesized, the base T was replaced by U, thus achieving the strand-specific library.

These double-stranded cDNA segments were end-repaired, and a single “A” base and ligation of adapters were added. The altered suitable components were then isolated through agarose gel electrophoresis to achieve the final paired-end cDNA libraries with a proper length (250–350 bp). Later, RNA-seq was completed on the Illumina NextSeq500 platform (Illumina, San Diego, CA, USA).

### Reads Processing and Assembly

The Cutadapt was utilized to filter low-quality reads (the base number of the mass value of Q ≤ 10 accounts for more than 20% of the entire read), the adaptor read, the N ratio of more than 5% of the reads from the raw reads to obtain the clean reads for further analysis. Then, the clean reads were blasted onto the human reference genome using Hisat2. Simultaneously, Q20 and Q30 were calculated. Three replicates were evaluated to minimize experimental errors, and filtered high-quality data was utilized for all downstream analyses. Raw Illumina sequences were uploaded to the National Center for Biotechnology Information Databank (NCBI) (accession number: PRJNA777752).

### DEGs Analysis

To analyze the expression of differential expression genes (DEGs), the quantification of DEGs is necessary. Thus, the gene expression was normalized as fragments per kilobase of transcript per million mapped reads (FPKM) (16). The adjusted *P*-values were accustomed following the Benjamini- Hochberg method for regulating the false discovery rate (FDR). DESeq software was used to detect DEG based on|log2FoldChange| > 1, with *P*-value < 0.05. The principal component analysis (PCA) of each sample was implemented on the significantly expressed genes. The correlation heat map was generated by the R package, and the volcano plots were accomplished by the OmicStudio tools (https://www.omicstudio.cn/tool).

### RNA-Seq Results Validation

14 DEGs Were Randomly Chosen to Validate the Sequencing Data by Quantitative Real-Time PCR (qRT-PCR). According to the Rigorous Principle, Every Primer for the Reaction Was Designed From the Primer 5.0 Software (Premier Biosoft International, Palo Alto, CA) According to the Thorough Guide (Table 1). Expression of β-Actin Was Utilized as an Internal Control (20). After RNA extraction with RNAsimple Total RNA Kit (TIANGEN, Beijing, China), the isolated RNA pellet was dissolved in 40 uL of ddH_2_O, and the concentrations and purity were evaluated by the Nanodrop ND2000 (Thermo Fisher). First-strand cDNA synthesis and qRT-PCR reaction were performed using FastKing One-Step RT-qPCR Kit (SYBR) (TIANGEN, Beijing, China). The qRT-PCR assays were set up on the LightCycler platform (Roche Diagnostics, Indianapolis, IN) with iTaq Universal SYBR Green Supermix Kit (Bio-Rad, USA). The 20 μL assay systems were as followed: 1μL of cDNA template, 0.8 μL of 10 μM primers, 10 μL SYBR Green Supermix, and 7.4 μl of ddH_2_O. The cycling conditions included a denaturation step at 95°C for 3 min, followed by 40 cycles at 95°C for 5 s and, and a final step of 60°C for 30 s. All the samples were run in triplicates, and the data were analyzed according to the 2^−ΔΔCt^ method.

**Table 1:** List of primers used in the present study.

**ID**	**Gene name**	**Sequence (5^**′**^-3^**′**^)**	**Length of product** **(bp)**
ENSG00000150593	PDCD4	GGGAGTGACGCCCTTAGAAG	108
		ACCTTTCTTTGGTAGTCCCCTT	
ENSG00000175387	SMAD2	CCGACACACCGAGATCCTAAC	125
		GAGGTGGCGTTTCTGGAATATAA	
ENSG00000123374	CDK2	CCAGGAGTTACTTCTATGCCTGA	90
		TTCATCCAGGGGAGGTACAAC	
ENSG00000137752	CASP1	TTTCCGCAAGGTTCGATTTTCA	54
		GGCATCTGCGCTCTACCATC	
ENSG00000125740	FOSB	GCTGCAAGATCCCCTACGAAG	249
		ACGAAGAAGTGTACGAAGGGTT	
ENSG00000033327	GAB2	ACAGTACCTACGACCTCCCC	109
		CTGGGCGTCTTGAAGGTGTA	
ENSG00000147889	CDKN2A	GGGTTTTCGTGGTTCACATCC	105
		CTAGACGCTGGCTCCTCAGTA	
ENSG00000124762	CDKN1A	TGTCCGTCAGAACCCATGC	139
		AAAGTCGAAGTTCCATCGCTC	
ENSG00000002330	BAD	CCCAGAGTTTGAGCCGAGTG	249
		CCCATCCCTTCGTCGTCCT	
ENSG00000116717	GADD45A	GAGAGCAGAAGACCGAAAGGA	145
		CACAACACCACGTTATCGGG	
ENSG00000130522	JUND	TCATCATCCAGTCCAACGGG	136
		TTCTGCTTGTGTAAATCCTCCAG	
ENSG00000118515	SGK1	GCAGAAGAAGTGTTCTATGCAGT	87
		CCGCTCCGACATAATATGCTT	
ENSG00000187514	PTMA	TCAGACGCAGCCGTAGACA	113
		GCATTCCCGTTAGCAGGGG	
ENSG00000079335	CDC14A	ACGCCCCTGAAGCCTACTT	126
		AGAAGAGGTCATAGTGCTCGAAG	

### Upstream Transcription Factors Prediction

The upstream transcription factors (TFs) of assessed DEGs were predicted utilizing FunRich software, a self-governing tool primarily for functional enrichment analysis of enriched genes of transcription factor pathways (21). The screened elevated and depressed DEGs were uploaded to calculate the top predicted TFs.

### Functional-Enrichment and Pathway Analysis

The Gene Ontology (GO) classification system (http://geneontology.org/) was applied to determine the possible functions of all the DEGs. The results were based on three independent ontologies: “biological process” (BP), “cellular component” (CC), and “molecular function” (MF). GO enrichment analysis blasted all the DEGs to terms in the GO database and screened out the significantly enriched GO terms of DEGs by a hypergeometric test with the complete genome as the background.

Kyoto Encyclopedia of Genes and Genomes (KEGG) (http://www.genome.jp/kegg) is a collection of databases that integrates data from genomic, chemical and system functions. Pathway enrichment analysis was conducted via the KEGG database to identify significantly enriched metabolic or signal transduction pathways, and the pathways with *P*-values < 0.05 were defined as significantly enriched pathways.

### Analysis of Hub Genes in Significantly Enriched Pathways

As a search platform, including the comprehensive protein-protein interaction (PPI) information, the STRING database delivers a PPI valuation and assemblies. In this exploration, the detected targets were uploaded to the STRING database (http://www.string-db.org/). PPI node pairs with a medium score ≥ 0.4 were filtered out. A Cytoscape-module identified as cytoHubba v0.1 (https://apps.cytoscape.org/apps/cytohubba) (22) was employed to determine the hub genes by evaluating node scores according to relevant algorithms (Degree Centrality, Betweenness Centrality, and Closeness Centrality).

### Western Blot

The treated HepG2 cells were washed with PBS and lysed with RIPA buffer (Sigma-Aldrich, USA) for 30 min until all the cell pellets were completely dissolved. After that, the cells were centrifuged at 14,000 rpm/min for 3 min and the supernatant was connected. Then the protein concentration was quantified by a BCA assay kit and 20–40 μg of each protein sample was separated by SDS-PAGE and located onto the PVDF membrane. After blocking with the blocking buffer (Beyotime, China), the membrane was incubated with a primary antibody (1/1,000 dilution) (Cell Signaling Technology, USA) at 4°C overnight, followed by secondary antibody (1/3,000 dilution) (Cell Signaling Technology, USA) for 1 h at room temperature. The signals were captured using a BeyoECL Sta Kit (Beyotime, China). β-actin was utilized as a reference control.

### Statistical Analysis

In this study, One-way ANOVA with a *t*-test at *p* < 0.05 was conducted by SPSS 18.0. The Origin 2019 (Origin Labs) software was used to formulate the figures. Except otherwise stated, mean ± SD (*n* ≥ 3) was used for data expression.

## Results

### Transcriptome Sequence and Assembly

After eliminating the short and redundant reads, 45,105,590 (99.47%), 42,016,916 (99.47%), 43,653,726 (98.87%) clean reads from the control groups (Control 1, Control 2, and Control 3); and 40,769,130 (98.61%), 46,280,800 (99.41%), and 40,711,164 (99.46%) clean reads from the LCA-treated groups (Treat A1, Treat A2, and Treat A3) were obtained (Table 2). The scores of Q20 and Q30 in every sample were higher. In addition, 38,111,391 (84.49%), 38,156,266 (90.81%), and 40,769,640 (93.39%) clean reads from the control groups; 37,349,404 (91.61%), 41,332,943 (89.31%), and 36,367,891 (89.33%) clean reads from the LCA-treated groups were obtained, which successfully matched to the reference genome. Thus, it showed that the transcriptome data prepared with good qualities was suitable for advanced bioinformatic evaluation.

**Table 2:** Statistical analysis of the RNAs Libraries.

**Sample**	**Raw reads**	**Clean Reads**	**Useful reads**	**Q20**	**Q30**	**Total Mapped**	**Mapped ration**
Control1	45,347,864	45,105,590	99.47%	95.71%	89.69%	38,111,391	84.49%
Control2	42,238,716	42,016,916	99.47%	97.36%	92.81%	38,156,266	90.81%
Control3	44,152,634	43,653,726	98.87%	97.27%	93.26%	40,769,640	93.39%
TreatA1	41,344,196	40,769,130	98.61%	96.68%	92.04%	37,349,404	91.61%
TreatA2	46,554,542	46,280,800	99.41%	97.28%	93%	41,332,943	89.31%
TreatA3	40,934,216	40,711,164	99.46%	97.29%	92.98%	36,367,891	89.33%

### Identification and Analysis of DEGs

**Figure 1:**
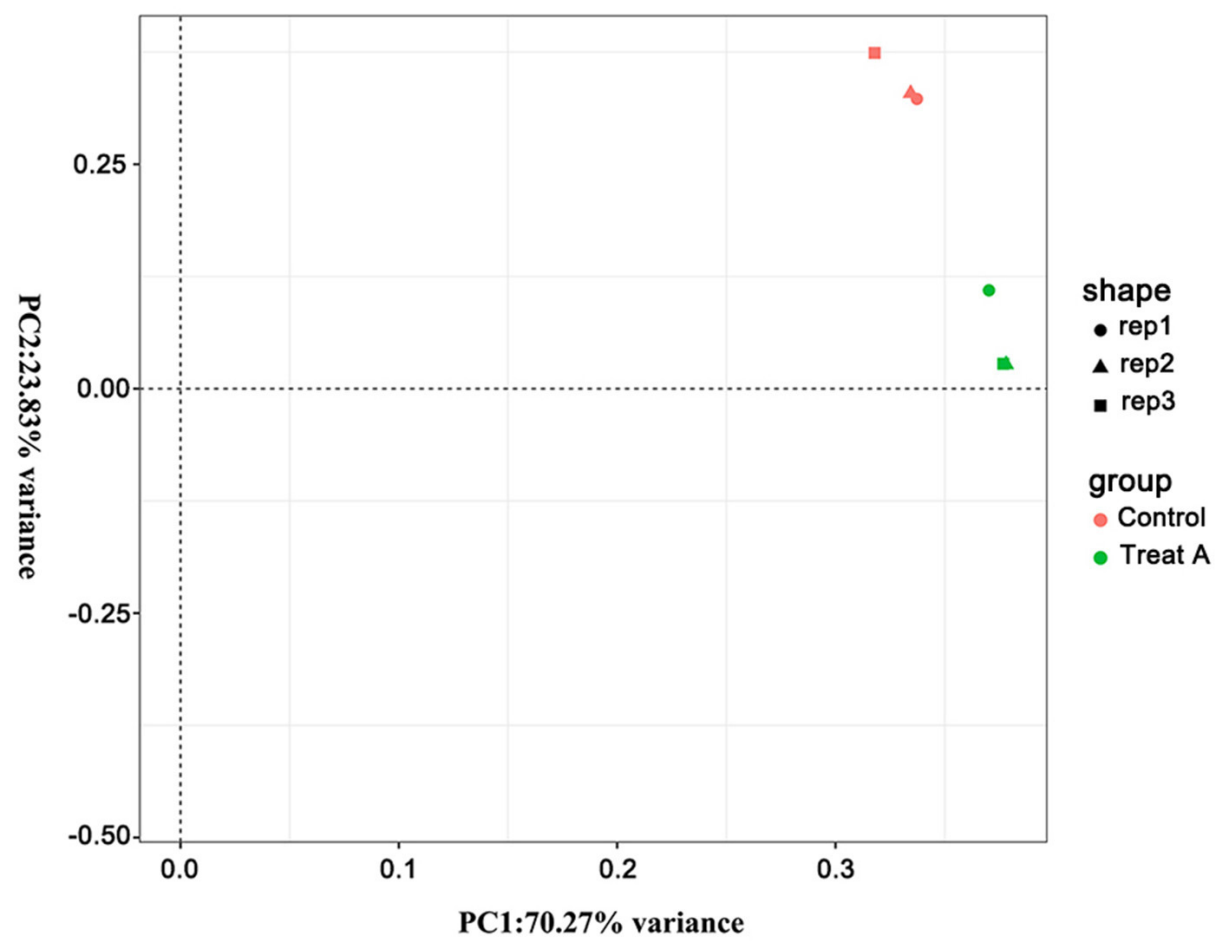
The PCA analysis of all samples. Different shapes represent different samples, different colors represent different groups.

**Figure 2:**
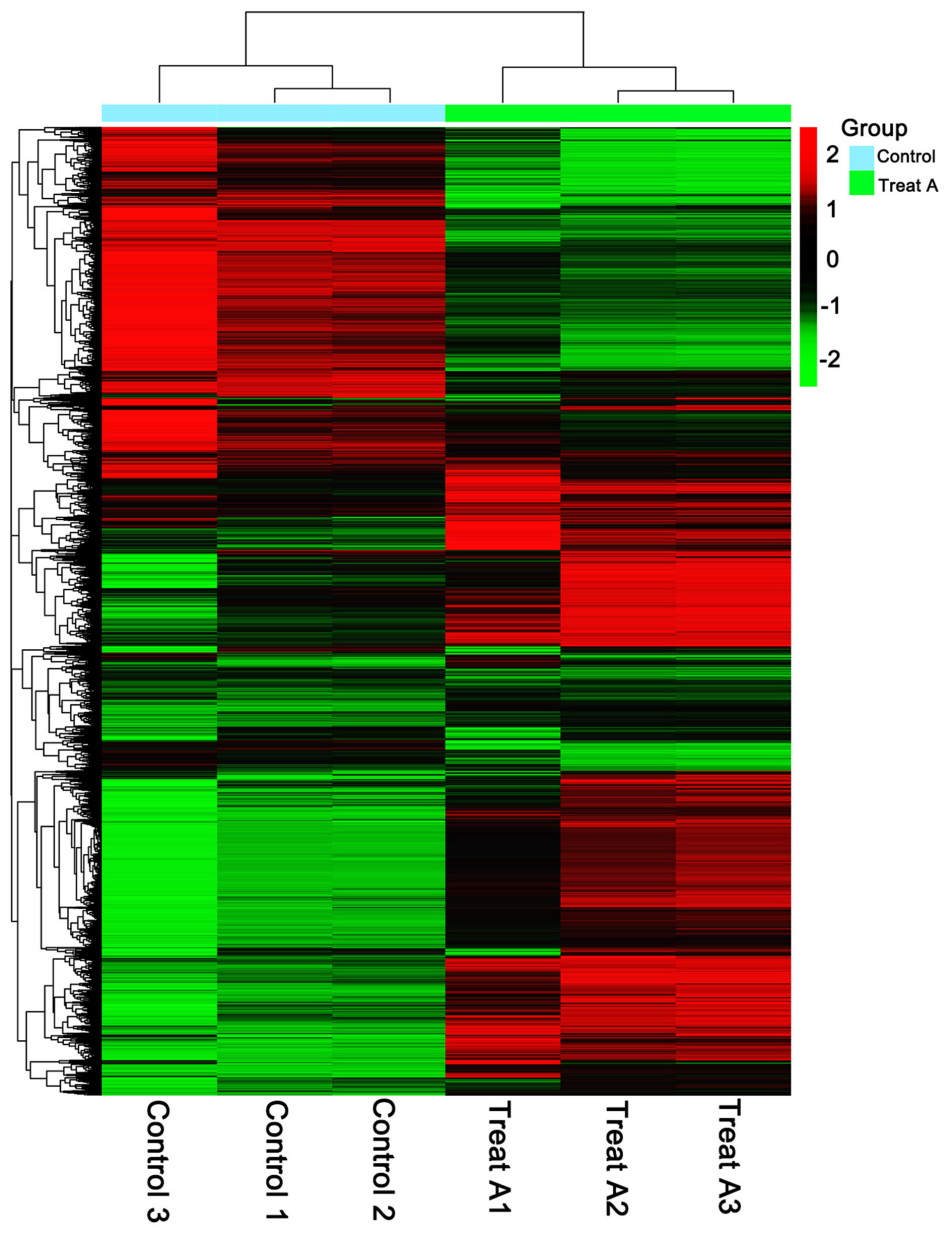
Heat map of lower (green) and higher (red) DEGs expression levels.

**Figure 3:**
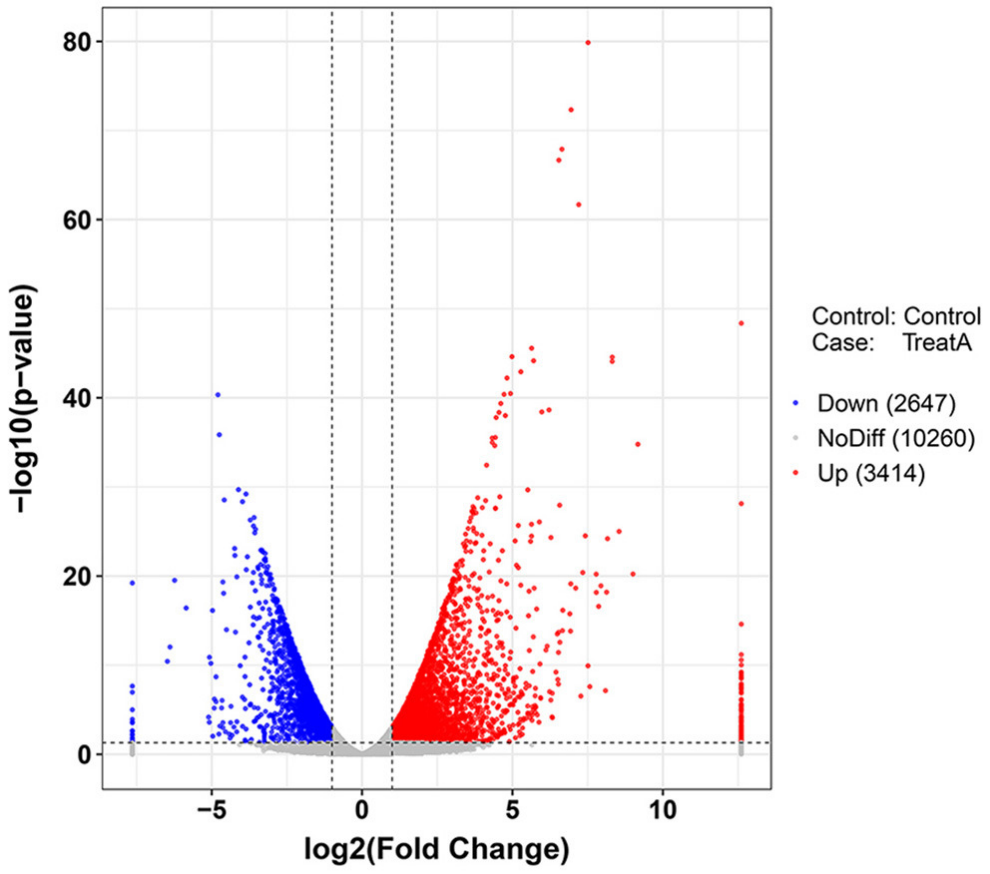
Volcano plot (*p* < 0.05) of down-regulated (blue) and up-regulated (red) DEGs expression levels. The blue bar indicates the percent of genes enriched to the transcript factors. The orange bar indicates the reference significance *p* = 0.05. The red bar indicates the significance of enrichment.

In Figure 1, the PCA analysis of these data demonstrated that each group was distinct from the other. In the heat map displayed in Figure 2, the genes were horizontally listed, one sample per column, red signified high expression genes, and green signified low expression genes. The heat map representation revealed a similarity in the transcriptomes within the three triplicate runs of the untreated group (Control 1, Control 2, and Control 3) and LCA treated group (Treat A 1, Treat A 2, and Treat A 3), respectively. However, as Figure 2 depicted, the expression of two different groups are distinguished clearly, consistent with the PCA analysis. DEGs analysis revealed that 6,061 genes were differentially expressed significantly between the LCA and control groups. As shown in the volcano map of DEGs (Figure 3), 3,414 genes were elevated and 2,647 genes were depressed.

### Validation of DEGs by qRT-PCR

**Figure 4:**
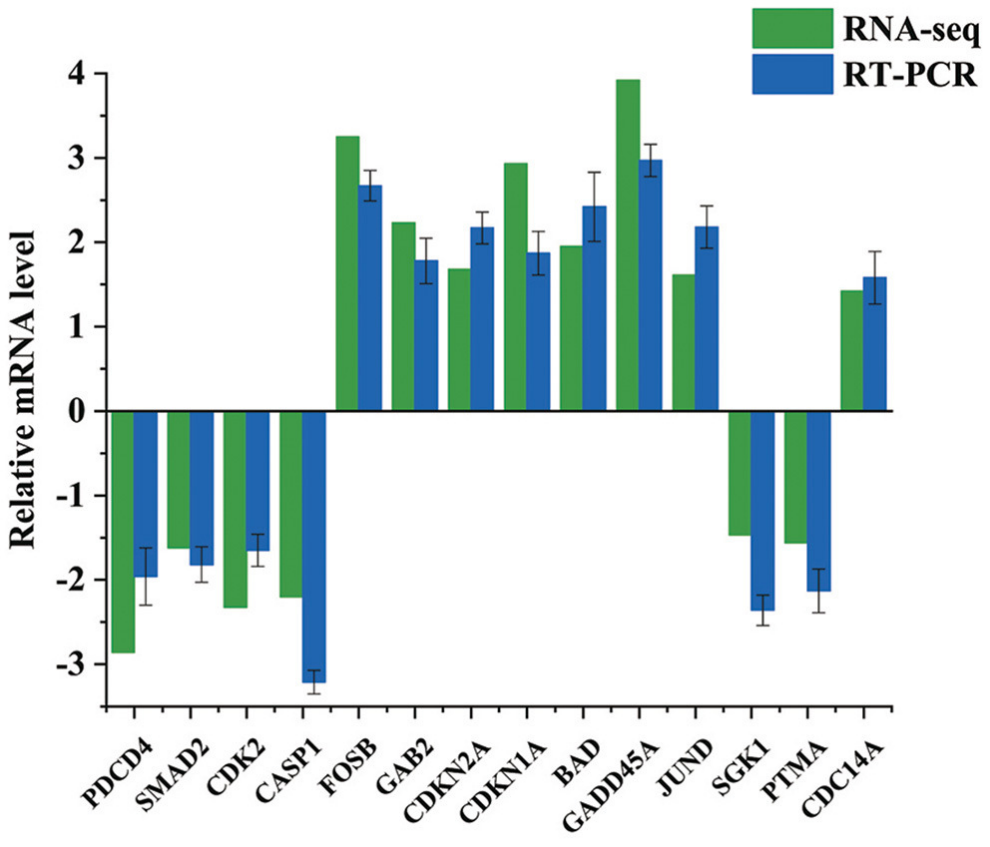
Validation of the DEGs by qRT-PCR. Bars show the relative expression level of DEGs. All the values were log2 transformed.

14 DEGs Were Selected for qRT-PCR Analyses to Confirm the RNA-Seq Results. According to the qRT-PCR Data, the Tendency of 14 Chosen MRNAs Covering eight up-Regulated and six Down-Regulated MRNAs Were Following the Transcriptome Results (Figure 4). For Example, the Expression Levels of PDCD4 From qRT-PCR and RNA-Seq Were−2.86 and−1.96, respectively. The Corresponding Expression Levels of BAD From qRT-PCR and RNA-Seq Were 1.95 and 2.42, respectively. Our Data Indicated That Above Results of Transcriptomics Data Were Reliable.

### Upstream Transcription Factors Prediction

**Figure 5:**
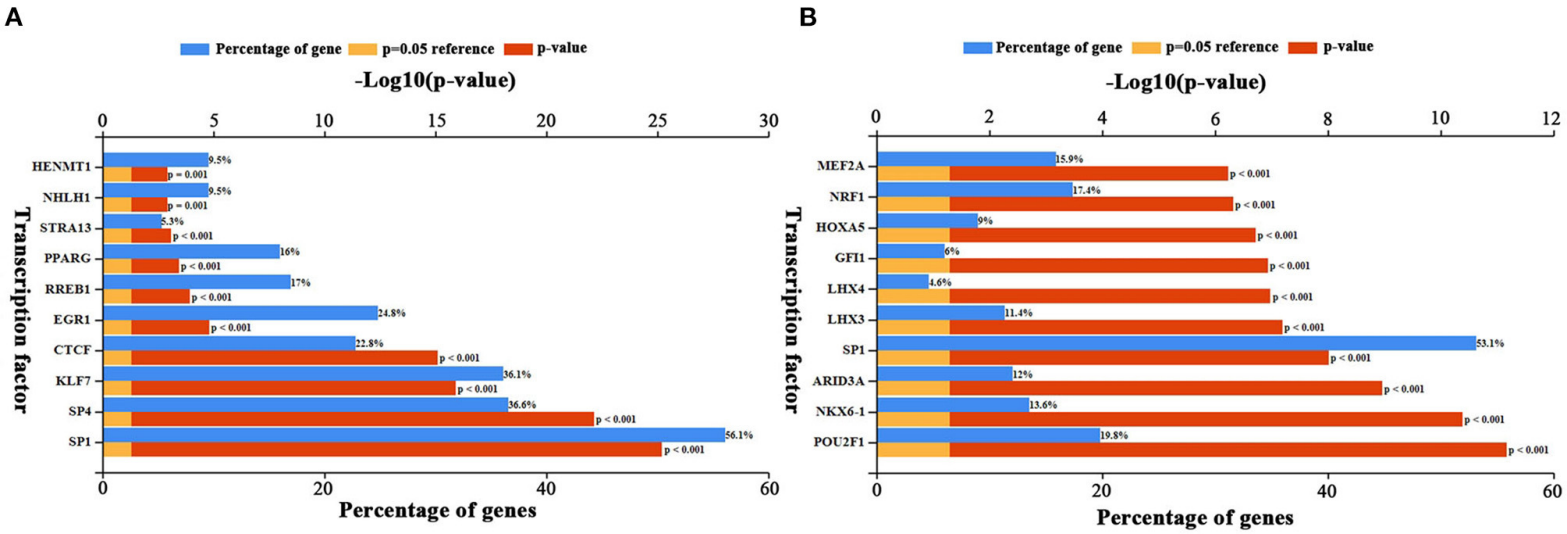
Predicted transcription factors of DEGs. **(A)** Transcription factors of up-regulated DEGs. **(B)** Transcription factors of down-regulated DEGs. The blue bars indicate the percentge of genes enriched to the transcription factor. The yellow bars indicate the reference significant enrichment (*P*-value = 0.05). The red bars indicate significant enrichment.

Upstream TFs of candidate elevated and depressed DEGs were predicted by FunRich. The top 10 TFs for increased and decreased DEGs were presented in Figures 5A,B, respectively. For increased DEGs, the top 10 TFs were HENMT1, NHLH1, STRA13, PPARG, RREB1, EGR1, CTCF, KLF7, SP4, and SP1. For decreased DE-miRNAs, the top 10TFs were MEF2A, NRF1, HOXA5, GFI1, LHX4, LHX3, SP1, ARID3A, NKX6-1, and POU2F1.

### GO Enrichment and Classification Analysis

The significantly enriched GO terms were showed in Table 3. These DEGs were significantly enriched in the cellular component organization or biogenesis (GO:0071840) with the *P*-value = 7.75E-19; cellular component organization (GO:0016043) with the *P*-value = 9.95E-19; cellular metabolic process (GO:0044237) with the *P*-value = 1.13E-16; metabolic process (GO:0008152) with the *P*-value =1.2E-16; primary metabolic process (GO:0044238) with the *P*-value = 1.82E-15 in the biological process (BP)-associated category. In the cell component (CC) -associated category, the most significant GO terms were intracellular part (GO:0044424) with the *P*-value = 9.83E-70; intracellular (GO:0005622) with the *P*-value = 4.59E-61; intracellular membrane-bounded organelle (GO:0043231) with the *P*-value = 1.38E-58; membrane-bounded organelle (GO:0043227) with the *P*-value = 1.03E-57 and intracellular organelle part (GO:0044446) with the *P*-value = 2.25E-56. Furthermore, the most significantly enriched in the molecular function (MF) -associated category were protein binding (GO:0005515) with the *P*-value = 6.21E-56; binding (GO:0005488) with the *P*-value = 3.67E-44; heterocyclic compound binding (GO:1901363) with the *P*-value = 5.73E-16; organic cyclic compound binding (GO:0097159) with the *P*-value =7.69E-16 and enzyme binding (GO:0019899) with the *P*-value =1.51E-13.

**Table 3:** The most significantly enriched GO terms.

**Category**	**ID**	**Description**	**Term *P*-value**
BP-associated category	0071840	Cellular component organization or biogenesis	7.75E-19
	0016043	Cellular component organization	9.95E-19
	0044237	Cellular metabolic process	1.13E-16
	0008152	Metabolic process	1.2E-16
	0044238	Primary metabolic process	1.82E-15
CC-associated category	0044424	Intracellular part	9.83E-70
	0005622	Intracellular	4.59E-61
	0043231	Intracellular membrane-bounded organelle	1.38E-58
	0043227	Membrane-bounded organelle	1.03E-57
	0044446	Intracellular organelle part	2.25E-56
MF-associated category	0005515	Protein binding	6.21E-56
	0005488	Binding	3.67E-44
	1901363	Heterocyclic compound binding	5.73E-16
	0097159	Organic cyclic compound binding	7.69E-16
	0019899	Enzyme binding	1.51E-13

### KEGG Pathway Analysis

**Figure 6:**
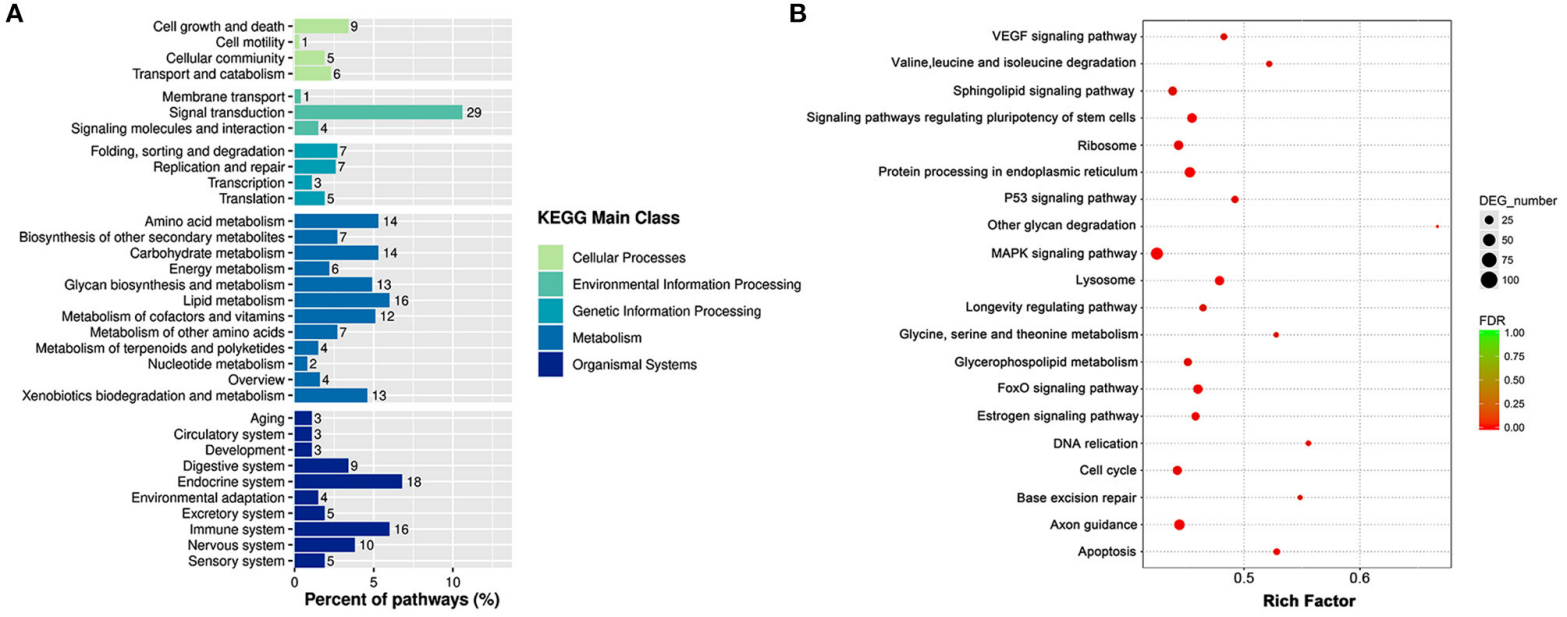
The KEGG enrichment analyses of DEGs. **(A)** Histogram of KEGG pathways based on the enrichment classification. **(B)** Scatter diagram of signal transduction pathways of KEGG.

According to the KEGG pathway analysis, the DEGs were enriched into 263 identified pathways. And the identified pathways were classified into 33 subclasses of five main categories. For example, four of them belonged to the cellular process, three of them were connected with environmental information processing, four were related to genetic information processing, 12 pertained to metabolism, and 10 were associated with organism systems. And the signal transduction was the most enriched sub classification as shown in Figure 6A. Correspondingly, to further acknowledge the most significant pathways, the bubble chart of Figure 6B exhibited the top 20 significantly enriched pathways. As it is depicted, the DEGs were enriched in pathways as the MAPK signaling pathway, the FoxO signaling pathway, the P53 signaling pathway, the cell cycle, and the apoptosis. A total of 102 DEGs were enriched in the MAPK signaling pathway, including 63 increased genes and 39 down-regulated genes. A sum of 102 DEGs was enriched in the FoxO signaling pathway, including 30 increased genes and 28 decreased genes.

### Analysis of Hub Genes in Significantly Enriched Pathways

**Figure 7:**
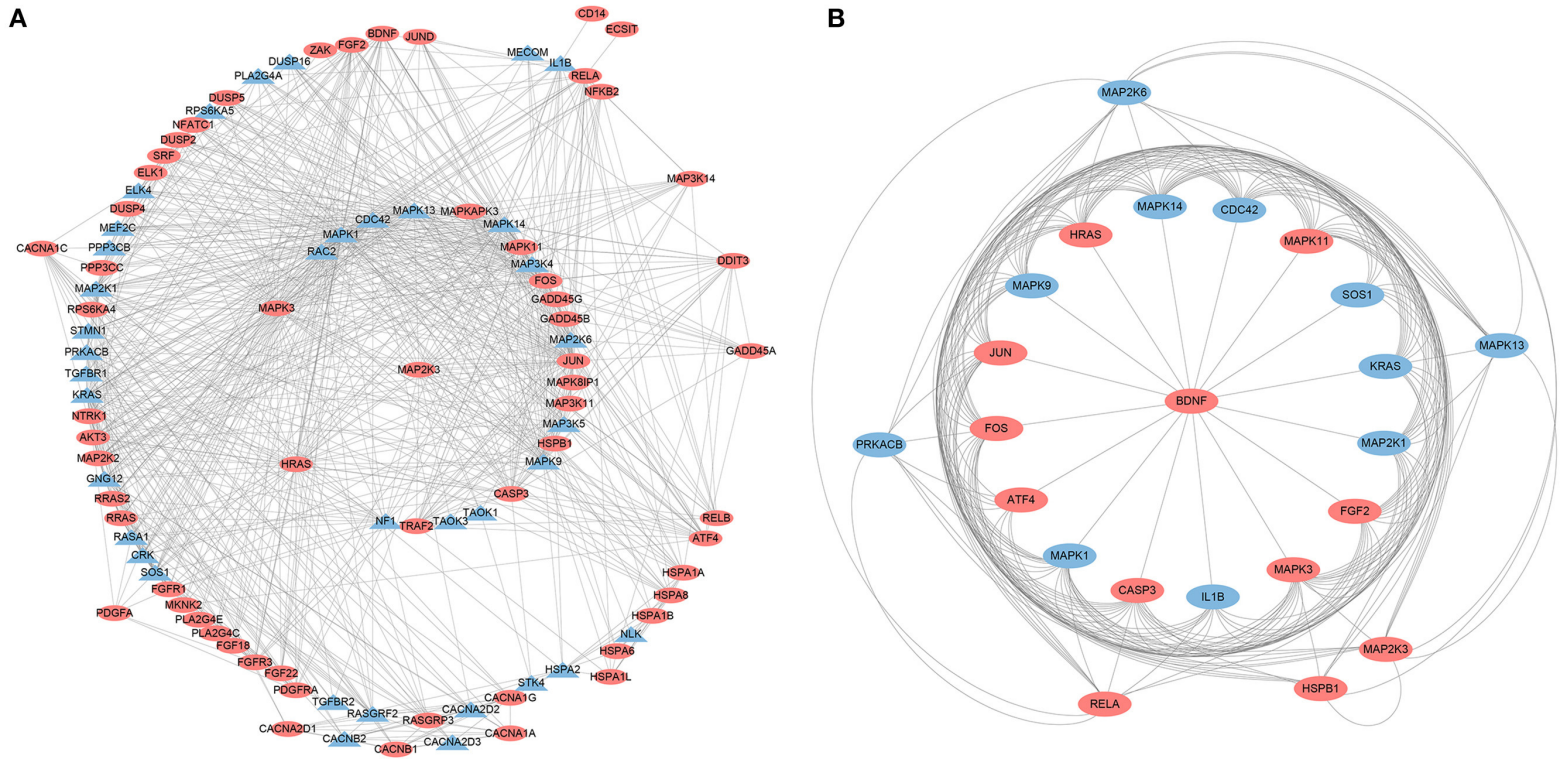
The PPI analysis of DEGs in the MAPK signaling pathways. **(A)** The entire PPI network of annotated interactions between genes. **(B)** A sub-network module for the hub genes. Red circles represent up-regulated genes, blue circles represent down-regulated genes.

**Figure 8:**
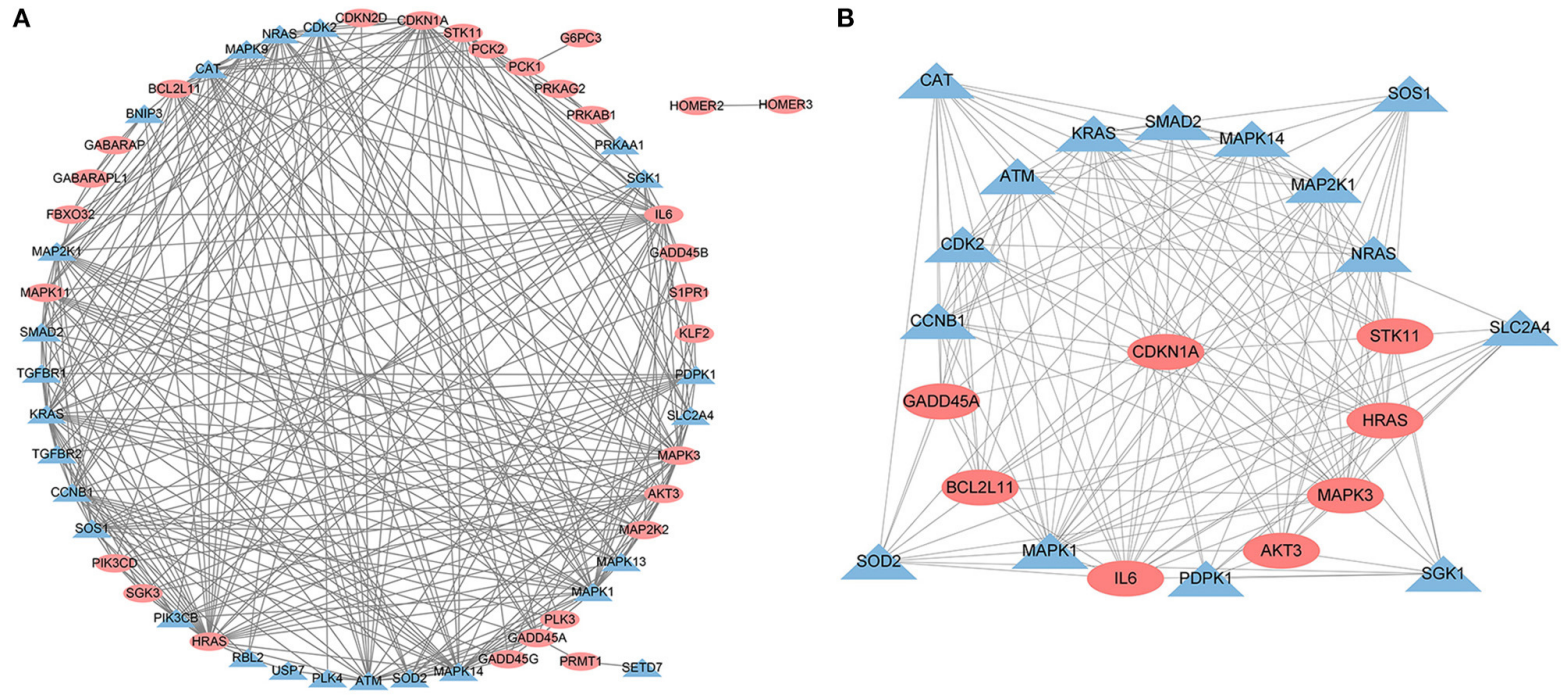
The PPI analysis of DEGs in the FoxO signaling pathways. **(A)** The entire PPI network of annotated interactions between genes. **(B)** A sub-network module for the hub genes. Red circles represent up-regulated genes, blue circles represent down-regulated genes.

A PPI network was developed for the DEGs that participated in the MAPK signaling pathway, including 97 connected nodes and 818 edges (Figure 7A). Through relevant topology calculation, the top 30 nodes were filtered. Later, 23 overlapped genes amongst these top 30 nodes were selected as hub genes (Table 4), including ATF4, BDNF, CASP3, CDC42, FGF2, FOS, HRAS, HSPB1, IL1B, JUN, KRAS, MAP2K1, MAP2K3, MAP2K6, MAPK1, MAPK11, MAPK13, MAPK14, MAPK3, MAPK9, PRKACB, RELA, and SOS1 (Figure 7B). The PPI network of DEGs in the FoxO signaling pathway, which included 55 connected nodes and 310 edges as illustrated in Figure 8A. For hub gene identification, the degree algorithm in the Cytohubba packages was used to identify the top 30 nodes. And 23 shared genes overlapped these 30 nodes were filtered as hub genes (Table 5), including AKT3, ATM, BCL2L11, CAT, CCNB1, CDK2, CDKN1A, GADD45A, HRAS, IL6, KRAS, MAP2K1, MAPK1, MAPK14, MAPK3, NRAS, PDPK1, SGK1, SLC2A4, SMAD2, SOD2, SOS1, and STK11 (Figure 8B).

**Table 4:** Top 30 nodes of PPI network in the MAPK signaling pathway.

**Name**	**Degree**	**Name**	**Betweenness**	**Name**	**Closeness**
MAPK1	63	MAPK3	1043.17	MAPK1	79.5
MAPK3	62	MAPK1	974.84	MAPK3	79
JUN	56	JUN	751.09	JUN	75.83
MAPK14	52	MAPK14	530.73	MAPK14	73.5
FOS	45	MAPK9	454.51	FOS	70.33
HRAS	42	HRAS	453.15	HRAS	68.67
MAPK11	42	PRKACB	437.33	MAPK11	68.33
MAPK9	40	KRAS	338.63	MAPK9	67.5
KRAS	38	FOS	322.86	KRAS	66.5
MAP2K1	37	CDC42	322.49	MAP2K1	65.83
CDC42	36	MAP2K6	322.36	CDC42	65.5
CASP3	34	CASP3	309.64	CASP3	64
MAPK13	33	HSPB1	304.13	MAPK13	63.17
MAP2K6	26	RELA	298.55	RELA	60
SOS1	26	IL1B	248.76	BDNF	59.83
FGF2	26	MAPK11	224.01	MAP2K6	59.67
PRKACB	26	MAP2K3	177.98	FGF2	59.67
MAP2K3	25	MAP2K1	170.58	PRKACB	59.5
RELA	25	GNG12	168.38	SOS1	59.17
BDNF	25	RASGRF2	165.73	MAP2K3	59
MAP2K2	22	RASGRP3	133.17	HSPB1	57.83
HSPB1	22	HSPA2	125.24	MAP2K2	57.33
ATF4	21	FGF2	120.29	ATF4	57.33
MAP3K5	20	BDNF	117.31	RAC2	57
RAC2	19	MAPK13	116.74	MAP3K5	57
NF1	19	SOS1	93.32	NF1	56
DDIT3	19	TRAF2	77.67	CRK	55.33
AKT3	18	CACNA1C	72.94	AKT3	55.17
TRAF2	18	ATF4	62.00	RASA1	55.17
IL1B	18	DDIT3	57.08	IL1B	55.17

**Table 5:** Top 30 nodes of PPI network in the FoxO signaling pathway.

**Name**	**Degree**	**Name**	**Betweenness**	**Name**	**Closeness**
HRAS	30	HRAS	290.91	HRAS	40.42
MAPK1	28	STK11	268.19	MAPK1	39.33
KRAS	27	GADD45A	219.54	KRAS	38.33
MAPK3	26	CDKN1A	212.26	IL6	38.25
IL6	26	IL6	203.74	MAPK3	38.08
CDKN1A	24	CAT	182.99	CDKN1A	37.25
MAPK14	23	MAPK1	181.31	MAPK14	36.83
MAP2K1	21	ATM	179.26	MAP2K1	35.58
NRAS	20	BNIP3	147.40	ATM	35.33
ATM	20	CCNB1	116.53	CCNB1	34.83
CCNB1	20	KRAS	107.30	NRAS	34.83
AKT3	18	PCK1	102	CAT	34.5
CAT	18	PRMT1	102	BCL2L11	33.67
STK11	17	MAPK3	101.49	STK11	33.58
BCL2L11	17	MAPK14	97.15	AKT3	33.42
SOS1	16	BCL2L11	70.22	CDK2	32.58
MAPK11	16	SLC2A4	65.13	SOS1	32.17
CDK2	16	SOD2	61.80	MAPK11	32.08
SMAD2	14	CDK2	61.00	SMAD2	31.33
GADD45A	13	FBXO32	58.89	GADD45A	31.17
MAP2K2	13	NRAS	43.09	SOD2	31
PDPK1	13	MAP2K1	41.70	PDPK1	30.75
SGK1	12	SGK1	39.76	MAP2K2	30.33
PIK3CB	12	PDPK1	37.25	SGK1	30
SOD2	12	AKT3	37.01	PIK3CB	29.5
MAPK9	11	SOS1	23.88	MAPK9	29.42
MAPK13	11	SMAD2	23.43	SLC2A4	29.42
SLC2A4	10	PCK2	13.97	MAPK13	29.42
TGFBR2	9	RBL2	13.73	TGFBR2	28.67
PIK3CD	9	SGK3	13.59	TGFBR1	27.67

### Detecting of DEGs in Significantly Enriched Pathways by Western Blot Assay

**Figure 9:**
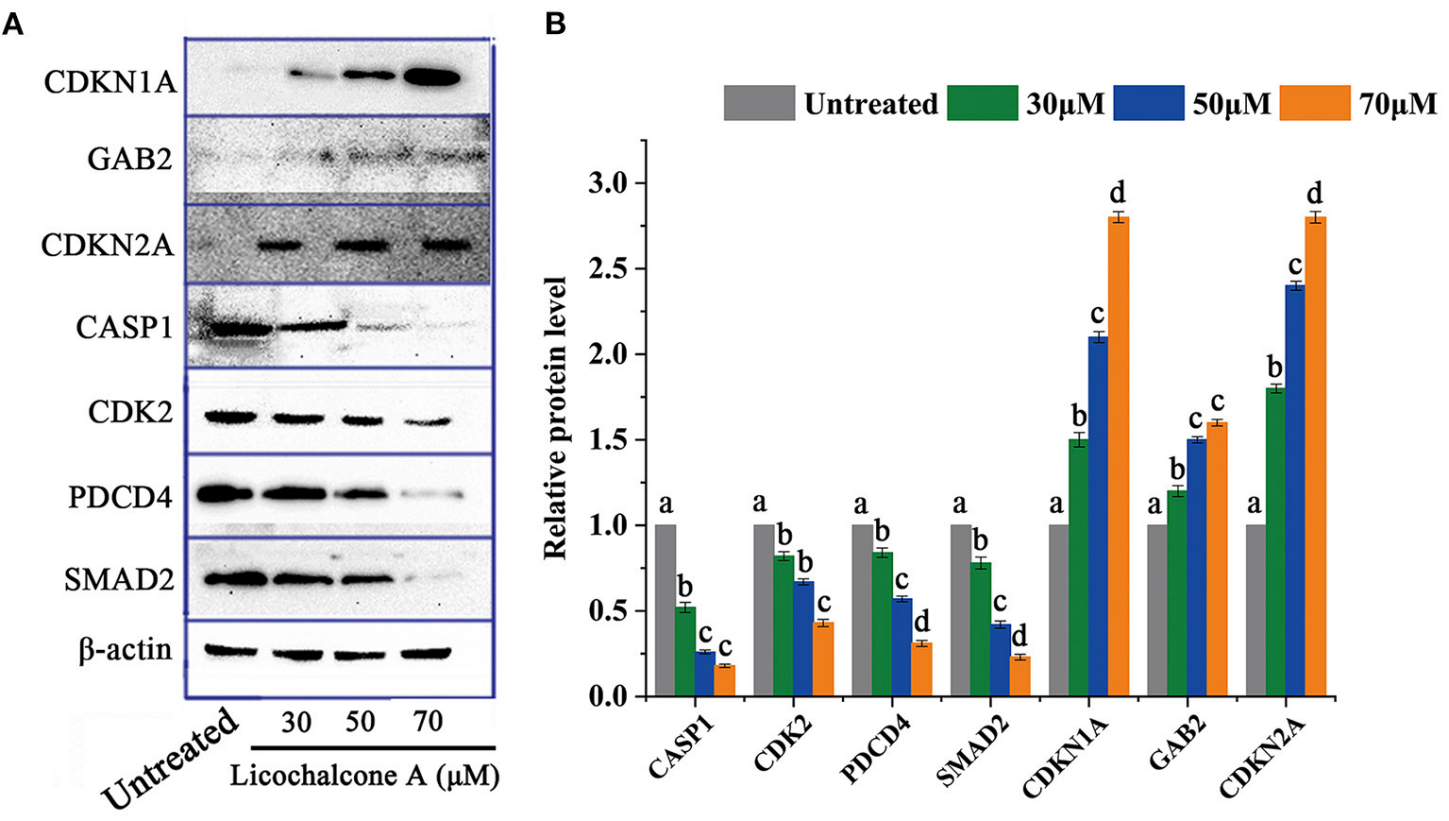
The protein level of DEGs in critical signaling pathways. **(A)** Protein expression profile. **(B)** Expression levels of related proteins. Significant differences between the two groups were denoted by different letters (*p* < 0.05).

Through transcriptome data analysis, some essential genes (CDKN1A, GAB2, CDKN2A, CASP1, CDK2, PDCD4, and SMAD4) related to significant pathways were screened and further analyzed. HepG2 cells were treated with different concentrations of LCA (0μM, 30 μM, 50 μM, and 70 μM) for 24h, and then the above genes were selected for protein level analysis. The protein expression of CDKN1A, GAB2, and CDKN2A was significantly up-regulated, while the protein expression of CASP1, CDK2, PDCD4, and SMAD2 were down-regulated (Figure 9).

## Discussion

Lately, HCC has appeared as a principal cause of cancer-related mortality. Though the death rate of HCC has decreased owing to better-quality medical environments, many people still suffer from it every year. *Glycyrrhiza* is a prospective resource for food and medical purposes with low toxicity. According to the Food and Drug Administration (FDA) declaration, the *Glycyrrhiza* root extract is generally recognized as safe. And the European Food Safety Authority (EFSA) panel also states that the licorice extract as a food additive is safe for the main human adult population up to 100 mg on daily basis (7).

LCA, recognized as a chalcone extracted from *Glycyrrhiza*, possesses anti-cancer abilities. Our previous study reported that LCA derived from *Glycyrrhiza* could restrain cell viability, induce cell cycle arrest and apoptosis, and elevate the cellular ROS levels in HepG2 (8). The underlying mechanism of the anti-cancer activities of LCA remains elusive. The acknowledgment of the transcriptome level in the progression of LCA-induced anti-cancer effects on Human hepatoma cell HepG2 which can illustrate the fundamental mechanism to develop new agents for HCC.

As an efficient molecular biology tool, RNA-seq delivers extraordinary details about transcriptional machinery of an organism (23). We screened all the transcripts of HepG2 cells stimulated by LCA. RNA-seq analysis found 6,061 DEGs between the LCA and control groups, including 3,414 elevated genes and 2,647 down-regulated genes. Based on the obtained DEGs, the TFs prediction, GO, and KEGG analysis, PPI networks were performed to investigate the effect of LCA on the HepG2 cells.

TFs are a group of proteins that can bind DNA recognition sites to govern genomic transcription. Various studies have indicated that gene expression can be regulated by TFs (24). Thus, we predicted the TFs that can restrict these DEGs. SP1, a zinc-finger TF that preferentially attaches to many GC-rich motifs of promoter region (25), can regulate the most screened DEGs expression. It can act as a critical factor to modulate the mRNA profile. For example, a recent study has proved that SP1 is involved in the PI3K/Akt signaling pathway, thereby contributing to tumor angiogenesis and hepatocellular development (26). EGR1, a member of TFs regulating cell proliferation and differentiation, can be rapidly and transiently induced by multiple factors, such as cytokines and mechanical stresses (27). It has been proved that EGR1 can regulate CBX8 to exhibit oncogenic activity in HCC (28). KLF7 can regulate cell growth and differentiation in multiple tissues and organs (29). KLF7 bound to the promoter of Ccdc85c aggravated the HCC progression. CTCF controls the metallothionein family's transcription activity in HCC, and editing CTCF binding sites provides a novel hint for cancer treatment (30). The POU2F1 transcription factor is one of the most critical regulatory proteins in humans and other mammals. The promoted expression of POU2F1 accelerates the growth and metastasis of HCC through the FAT1 signaling pathway (31). HOXA5, which encoded a DNA-binding transcription factor, can control gene expression, cell morphogenesis, and differentiation in various cancer cells. A previous study found that HOXA5 inhibits invasion and metastasis of HCC by regulating the expression of UBC9 (32). MEF2A altered histone acetylation seems to play a crucial part in regulating gene transcription and carcinogenesis. The over expression of MEF2A activated hepatic cells participate in the pathogenesis of HCC (33). GFI1 interacts with other cofactors to block the transcription process in cells. In HCC, GFFI can mediate long non-coding RNA to restrain the cancer cell metastasis (34).

GO is a globally standardized functional category system of genes that delivers an updated controlled vocabulary to illustrate the properties of genes in organisms comprehensively. The GO databank comprises three categories of genes as MF, CC, and BP. From the results, the BP aspect was mainly enriched in cell metabolism and compound synthesis types. And as for the CC category, the genes were primarily enriched in the nucleus aspect. Besides, protein binding, ATP binding, DNA binding, and other functions were found in the MF category. In brief, the GO analysis correlates the DEGs with regulating the metabolic process, intracellular structure, and binding activities related to cancer progression.

KEGG is a utility database to exhibit complex functional and biological systems, generating statistics from the molecular level. One of the most prominent KEGG pathway analysis features is integrating the genome, chemistry, and system functions and visualizing the network of intermolecular interactions through powerful graphics functions. In the current study, the KEGG analysis specified that these DEGs were significantly enriched in the signal transduction category. And the MAPK signaling pathway and the FoxO signaling pathway are identified as significant pathways. The MAPK signaling pathway performs a crucial part in extracellular signal transduction into cellular responses. The MAPK signaling pathway amplifies and integrates signals from various stimuli and triggers appropriate physiological responses, including inflammation and apoptosis in mammalian cell proliferation, differentiation, and development (35). Many phytonutrients are reported to suppress metastasis through the MAPK signaling pathway in HepG2 cells (36). KEGG analysis in the present study identified 102 DEGs in connection with the MAPK signaling pathway. The PPI network depleted 23 hub genes in the MAPK signaling pathway, such as MAPK1, MAPK3 MAPK11, MAPK13, MAPK14, ATF4, BDNF, CASP3, etc. MAPK1 functions as a negative regulator for MAPKs and reduces oxidative stress involved in mitochondrial damages and cell death activation. And ATF4 can block the MAPK1 expression (37). MAPK3 was one of the first identified MAP kinases, participated in the cell-cycle process and controlled cell survival and apoptosis by regulating both the expression and the activity of proapoptotic proteins (38). MAPK11, MAPK13 and MAPK14 were members of p38 MAPKs (39). The p38 MAPKs was one of the four major MAPKs cascades and played an important role in cellular responses evoked by extracellular stimuli such as proinflammatory cytokines or physical stress. The p38 MAPKs also had high sequence homology with CDK family members, thus could cooperate with CDKs, and regulate the cell cycle. For liver tumorigenesis, the p38 MAPKs were essential for the cancer cell cycle progression and are identified as the promising therapeutic targets for the disease (40). BDNF participates in the activation of MAPKs and is a novel functional protein in HCC (41). CASP3 is the upstream binding factor in the MAPK signaling pathway. Anticarcinogen stimulates apoptosis of HCC through damaging mitochondria and activating CASP3 (42). Recent reports have revealed novel roles of the FoxO signaling pathway in cell proliferation and tumorigenesis. Many phytochemicals are reported to display their anti-cancer activities by mediating the FoxO signaling pathway. For example, the extract of *Aegiceras corniculatum* induced apoptosis on human colorectal cancer via the FoxO signaling pathway (43). It is renowned that the FoxO signaling pathway exerts an enormous function in HCC etiology. In this study, 26 hub genes related to the FoxO signaling pathway were screened out, such as AKT3, CDK2, CDKN1A, GADD45A, IL6, etc. AKT3 is often hyper activated in cancer disease and functions as a biomarker for therapy strategies. The disorder of AKT3 can promote spontaneous HCC (44). GADD45A acts as a stress sensor for cell damage and forms a complex with CDKN1A to administer the cell cycle process. IL6 is a pro-inflammatory cytokine response to phytochemicals (45). In HCC, the dysregulation of IL6 is responsible for anticarcinogen resistance (46).

The protein level of essential genes that participated in significant pathways was consistent with the relevant research. In this study, the protein level of CDKN1A, GAB2, and CDKN2A were elevated while the others were decreased. CASP1, as a vital member of the Caspases family, is involved in the MAPK signaling pathway, cell apoptosis, and other signaling pathways. Studies have shown that abnormal expression of CASP1 can induce cell apoptosis (47). CDK2 in cyclin, as an essential component of cell cycle mechanism, can form CDK2/cyclin E complex with cyclin E, and it is mainly active CDK2/cyclin A complex and phosphorylated E2F in the S phase of the cell cycle and remains in the nucleus in the form of CDK2 in G2 phase. A previous study revealed that phytochemicals could target the CDK2 to treat HCC (48). PDCD4, also known as a programmed cell death protein, participates in the apoptotic signaling pathway and acts as an inhibitor of tumorigenic transformation. For HCC, the dysregulated PDCD4 is testified to relate to the metastatic potential of cells (49). SMAD2 are critical components of intracellular pathways, which participate in many signal pathways such as the FoxO signaling pathway, cell cycle, etc. SMAD2 targets various DNA-binding proteins and regulates cell transcriptional response. The activation of SMAD2 can induce the epithelial-mesenchymal transition and invasion in HCC (50). Tumor suppressor gene CDKN1A, also known as p21, inhibits cell cycle progression, participates in the FoxO signal pathway, cell cycle, and p53 signal pathway. CDKN1A forms heterotrimeric complexes with cycle-dependent kinases. When CDKN1A is combined with CDK2, it can inhibit kinase activity and block the progress of the cell cycle. Some studies have shown that it can promote cell senescence, and the expression of CDKN1A in cancer cells often changes. The silencing of CDKN1A can promote the cell cycle, migration, and epithelial-mesenchymal transition progression of HCC (46). CDKN2A, known as P16, is a classical tumor inhibitor gene in various types of cancers. It regulates the cell cycle and the p53 signaling pathway. The reactivation of P16 exhibits antitumor potency in HCC (51). GAB2 is identified as an oncogene and binds to cell membrane receptors and downstream effectors to accelerate cell cancer. GAB2 mediates the process of HCC by integrating multiple signaling pathways (52).

## Conclusion

In conclusion, this study is the first effort to evaluate the effect of LCA on the transcriptome profile for HepG2 cells. Based on an inclusive analysis of DEGs, TFs enrichments, GO, and KEGG analysis elucidated that LCA induced the anti-cancerous activity on HepG2 cells, in which the MAPK signaling pathway and the FoxO signaling pathway played an essential role. The protein level of essential genes that participated in significant pathways was consistent with the transcriptome data. These findings implied that LCA could be implemented as a promising functional food ingredient and assist therapy in HCC, promoting a comprehensive view of its anti-cancer effects. The RNA-sequencing data at the high-throughput platform provided potential biomarkers to discover novel therapeutic targets for HCC.

## Data Availability Statement

Raw Illumina sequences were uploaded to the National Center for Biotechnology Information Databank (NCBI) (accession number: PRJNA777752).

## Author Contributions

JW: investigation, conceptualization, writing (original draft), and funding acquisition. BW: investigation, conceptualization, and validation. KT: writing (review and editing). K-XL and C-YW: software and formal analysis. Z-JW: supervision and funding acquisition. All authors contributed to the article and approved the submitted version.

## Funding

This study was supported by the Hefei University Scientific Research and Development Fund (20ZR09ZDB) and the talent fund of Hefei University (20RC48).

## Conflict of Interest

The authors declare that the research was conducted in the absence of any commercial or financial relationships that could be construed as a potential conflict of interest.

## Publisher's Note

All claims expressed in this article are solely those of the authors and do not necessarily represent those of their affiliated organizations, or those of the publisher, the editors and the reviewers. Any product that may be evaluated in this article, or claim that may be made by its manufacturer, is not guaranteed or endorsed by the publisher.

## Abbreviations

LCA: Licochalcone A
HCC: Hepatocellular carcinoma
RNA-seq: RNA sequencing
DEGs: Different expression genes
FPKM: Fragments per kilobase of transcript per million mapped reads
TFs: Transcriptional factors
qRT-PCR: Quantitative real-time PCR
GO: Gene Ontology
KEGG: Kyoto Encyclopedia of Genes and Genomes
PPI: Protein-protein interaction
BP: Biological process
CC: Cell component
MF: Molecular function
PCA: Principal component analysis
FDA: Food and Drug Administration
NCBI: National Center for Biotechnology Information Databank.
